# Loss plus adversity: posttraumatic stress and prolonged grief three years after the 2023 Türkiye earthquakes

**DOI:** 10.3389/fpsyt.2026.1983790

**Published:** 2026-09-28

**Authors:** Beyza Erdogan Akturk, Omer Kaptanoglu, Meltem Insel Kaptanoglu

**Affiliations:** 1 Psychiatry, Tarsus State Hospital, Mersin, Türkiye; 2 Department of Psychiatry, Psychiatry, Necip Fazil City Hospital, Kahramanmaraş, Türkiye

**Keywords:** bereavement, disaster mental health, earthquake, natural disaster, posttraumatic stress, prolonged grief disorder, traumatic adversity

## Abstract

Most mental health research following the 6 February 2023 Kahramanmaraş earthquakes has focused on community samples assessed within the first two years, leaving longer-term trauma- and loss-related psychopathology less well characterized. In this cross-sectional study, 149 adults who directly experienced the earthquakes were assessed at a psychiatry outpatient clinic in the affected region approximately three years after the disaster. Posttraumatic stress, prolonged grief, meaning attributed to loss, and rumination were assessed by self-report, while depression was clinician-rated using the Hamilton Depression Rating Scale. Grief-specific analyses were restricted to 64 participants who had lost a family member or close friend. Overall, 34.9% had elevated depressive symptoms, 23.5% met the screening threshold for posttraumatic stress, and 15.6% of those with a close loss met the algorithmic criteria for probable prolonged grief disorder. Among bereaved participants, those reporting additional earthquake-related adversity had substantially higher posttraumatic stress than those without additional adversity (43.0 vs. 16.7; Hedges g = 1.43, 95% CI [0.93, 1.94]) and greater grief severity (30.5 vs. 18.4; g = 0.97, 95% CI [0.45, 1.48]). Additional adversity remained associated with grief severity after adjustment for posttraumatic stress and depression. By contrast, posttraumatic stress was only modestly higher among participants with bereavement alone than among those with neither bereavement nor additional adversity, and the difference was not statistically significant (g = 0.30, 95% CI [−0.10, 0.69]); however, the confidence interval did not exclude a moderate effect. In secondary analyses, the association between brooding and posttraumatic stress was weaker among bereaved participants, and meaninglessness showed an incremental association with grief beyond posttraumatic stress and depression. Both findings varied across sensitivity analyses and should be regarded as preliminary. Three years after the earthquakes, symptom burden was substantial and was highest when bereavement co-occurred with additional disaster-related adversity.

## 1 Introduction

On February 6, 2023, two major earthquakes struck 11 provinces of Türkiye, killing more than 50,000 people and exposing millions to life threat, destruction, displacement, and the loss of loved ones (1). Disasters of this magnitude impose a dual burden of traumatic exposure and bereavement (2), and both outcomes can remain clinically significant for years after the acute phase (3). Although prolonged grief disorder (PGD) is now recognized in ICD-11 (4) and DSM-5-TR (5), reported rates after disasters vary substantially with the population studied, the assessment method, and the time since loss (6, 7). Research on the 2023 Türkiye earthquakes has largely relied on nonclinical cross-sectional samples assessed during the early post-disaster period, including community-based populations (8) and university students (9). To our knowledge, prolonged grief has not yet been examined in a clinical sample approximately three years after these earthquakes.

A key unresolved question is which components of a disaster are more closely associated with posttraumatic stress and prolonged grief. In disasters, traumatic adversities such as threat to life, injury, witnessing death, and deprivation often occur alongside the loss of a loved one. Posttraumatic stress and prolonged grief are related but distinct constructs (10, 11) with partly different risk profiles (12). Much of the direct evidence on this question comes from studies of the 2004 tsunami, in which direct exposure was more closely associated with posttraumatic stress, whereas characteristics of the loss were more predictive of prolonged grief (13); within that literature, findings on the association between direct exposure and grief severity were inconsistent (14, 15). Studies of other disasters point in a similar direction. Among bereaved survivors of the 2008 Sichuan earthquake, physical injury and terrifying earthquake experiences were associated with complicated grief, but these associations were no longer significant after posttraumatic stress symptoms were taken into account (16). After the September 11 attacks, complicated grief was associated with features of the loss, including the death of a child and the deceased having died at the World Trade Center, but not with the survivor’s own location during the attacks (17). Following the 2011 Great East Japan Earthquake, bereavement remained associated with posttraumatic stress symptoms after adjustment for house damage and other disaster-related exposures (18). However, few studies have examined bereavement and additional disaster-related adversity together in relation to both posttraumatic stress and grief, particularly in clinical samples assessed years after an earthquake.

The distinction between loss- and exposure-related pathways also raises the possibility that the links between psychological processes and symptoms may differ by bereavement status. We therefore examined two candidate processes alongside these stressor components. Repetitive negative thinking is a strong and consistent correlate of posttraumatic stress (19, 20). Longitudinal evidence suggests, however, that the strength of this association may vary across trauma types (21), while rumination following bereavement has been proposed to serve, at least in part, an avoidant function (22, 23). These findings suggest that the association between repetitive negative thinking and posttraumatic stress may differ according to bereavement status. Whether bereavement modifies this association among individuals exposed to the same disaster, however, remains unclear.

Meaning reconstruction models place difficulty integrating a loss into one’s sense of self and understanding of life at the center of problematic grief (24). Difficulties in meaning-making have been associated with greater grief severity (25), and meaninglessness has been identified as a central feature in symptom networks of prolonged grief (26). However, overlap in item content between measures of meaninglessness and prolonged grief may inflate the observed association. We therefore examined whether meaninglessness was associated with grief independently of other concurrent symptoms and assessed the extent to which this association depended on overlapping grief items.

In this study, we examined the associations of bereavement, additional earthquake-related adversity, and psychological processes with posttraumatic stress and prolonged grief in 149 adults assessed at a psychiatry outpatient clinic approximately three years after the earthquakes. Grief-specific analyses were restricted to 64 participants who had lost a family member or close friend. We hypothesized that additional adversity would be more strongly associated with posttraumatic stress than bereavement itself, and that symptom burden would be greatest among participants who experienced both bereavement and additional adversity. As secondary hypotheses, we expected that the association between repetitive negative thinking and posttraumatic stress would differ by bereavement status, and that, among those who had experienced a close loss, meaninglessness would be associated with prolonged grief beyond posttraumatic stress and depression.

## 2 Materials and methods

### 2.1 Participants and procedure

Data were collected between January 1 and March 31, 2026, at the psychiatry outpatient clinic of a training and research hospital in the earthquake-affected region. During this period, 257 adults who had directly experienced the Kahramanmaraş earthquakes of February 6, 2023 were assessed for eligibility. Inclusion criteria were age 18–65 years, direct exposure to the earthquakes, presentation to the clinic during the study period, ability to provide informed consent, and sufficient proficiency in Turkish to complete the assessment. Exclusion criteria were a psychotic or bipolar disorder, a neurologic disorder or intellectual disability that could interfere with the assessment, or communication difficulties that prevented completion of the study procedures. Ongoing psychiatric follow-up or treatment was not an exclusion criterion. Of the 257 individuals assessed, 43 were excluded because of clinical exclusion criteria and 11 because of communication difficulties. Of the 203 eligible individuals, 45 declined participation and 9 withdrew after providing consent. The final sample comprised 149 participants.

The outpatient sample was heterogeneous and included individuals presenting for an initial psychiatric evaluation, ongoing outpatient follow-up or treatment, or a medical-board assessment. Based on retrospective estimates by the assessing psychiatrists, approximately one third of participants presented for a medical-board assessment; however, reason for presentation was not recorded at the individual level, and this estimate should therefore be considered approximate. Psychiatric diagnoses not covered by the exclusion criteria, treatment status, and psychotropic medication use were likewise not systematically recorded. Accordingly, symptom frequencies were interpreted as characteristics of this clinical sample rather than as estimates of population prevalence.

Seventy-five participants (50.3%) reported at least one earthquake-related bereavement; 64 had lost a family member or close friend, whereas 11 had lost only a more distant friend. Analyses of the full sample (N = 149) used any earthquake-related bereavement as the grouping variable (n = 75), whereas grief-specific analyses were restricted to participants who had lost a family member or close friend (n = 64). The analytic sample used for each model is specified in the relevant section.

The study was conducted in accordance with the Declaration of Helsinki and was approved by the Toros University Scientific Research and Publication Ethics Committee on November 24, 2025 (decisions 09/170 and 09/171). Two concurrently approved protocols addressing grief/meaning and rumination/posttraumatic stress were combined in the present report to avoid fragmented publication of findings from the same sample. All participants provided written informed consent, and the data were anonymized before analysis. All measures were completed in a single session. The psychiatrist administered the HAM-D first. Participants then completed the self-report questionnaires in a fixed order, beginning with the sociodemographic and earthquake-related items (including bereavement and additional adversity), followed by the PG-13 and RRS-SF, and ending with the GMRI and IES-R. The self-report questionnaires took approximately 30 minutes. Because the questionnaire order was not counterbalanced, order effects and fatigue cannot be excluded. Participants received no financial compensation, and clinical support was provided when needed. The study was reported in accordance with the STROBE guidelines.

### 2.2 Measures

Validated Turkish versions of all instruments were used. Internal consistency coefficients are reported in **Supplementary Table 1**; reliability estimates for grief-specific measures were calculated in the close-loss subsample.

#### 2.2.1 Prolonged grief

Prolonged grief was assessed with the Prolonged Grief Scale (PG-13) among participants who had lost a family member or close friend (27, 28). Symptom severity was calculated as the sum of 11 symptom items (α = .96). Probable prolonged grief disorder was determined using the A–E algorithm applied in the Turkish validation study, including duration and functional impairment criteria. This algorithm is based on the original PG-13 criteria and does not represent a direct operationalization of DSM-5-TR or ICD-11 diagnostic criteria (29).

#### 2.2.2 Meaning attributed to loss

The six-item Emptiness and Meaninglessness subscale of the Grief and Meaning Reconstruction Inventory (GMRI) was used (α = .82) (30, 31). Participants who reported more than one loss were instructed to answer with reference to the person to whom they had felt closest. The PG-13 and GMRI were completed with reference to the same loss.

#### 2.2.3 Posttraumatic stress

Posttraumatic stress symptoms were assessed with the Impact of Event Scale–Revised (IES-R) completed with reference to the earthquakes (32, 33). The total score was used (α = .97). A cutoff score of ≥33 was used descriptively to identify participants with symptom levels in the probable-PTSD range and was not treated as a diagnostic classification.

#### 2.2.4 Depression

Depressive symptoms were assessed with the 17-item Hamilton Depression Rating Scale (HAM-D), administered by a single psychiatrist (34, 35). The total score was used, with scores ≥8 considered to indicate at least mild depressive symptom severity (36).

#### 2.2.5 Rumination

The short form of the Ruminative Responses Scale (RRS-SF) was used to derive brooding (α = .90) and reflection (α = .86) subscale scores (37, 38).

#### 2.2.6 Additional earthquake-related adversity

Additional burden beyond the shared earthquake experience was assessed with a single multiple-response item asking whether participants had experienced any further earthquake-related adversity; response options were serious injury, entrapment under rubble, witnessing death, difficulty obtaining food, and shelter problems, and participants were instructed to mark all that applied. The item also included an explicit “none” option, and because the assessment was administered face-to-face with an investigator present, unmarked options were coded as absent. The count represents the number of adversity types endorsed (0–5) and does not weight experiences according to their severity. Five participants who marked both “none” and at least one adversity type were coded according to the types endorsed, consistent with the rule applied to the bereavement item, and entries in a free-text “other” field (n = 8) were reviewed and not incorporated into the count, as none corresponded to the five adversity types. Because serious injury and entrapment under rubble were rare (one and two participants, respectively), the count in practice primarily reflected witnessing death, difficulty obtaining food, and shelter problems. A three-item count excluding the two rare types was examined in sensitivity analyses, and the individual adversity types were examined in an exploratory analysis (**Supplementary Table 2**). Post-earthquake migration was recorded separately; additional adjustment for migration did not materially change the findings.

#### 2.2.7 Sociodemographic characteristics

Age, sex, educational attainment, and marital status were recorded

### 2.3 Statistical analysis

Analyses were conducted in R 4.6.0. All tests were two-sided with α = .05, and there were no missing data for variables included in the analyses. Bereaved and non-bereaved participants were compared using Welch’s t tests for continuous variables, χ² tests for categorical variables, and Fisher’s exact tests for individual adversity types. Bias-corrected Hedges g and 95% confidence intervals were reported for continuous group differences. Pearson correlations were used to examine bivariate associations.

Ordinary least squares regression was used throughout. IES-R scores were square-root transformed and PG-13 scores log-transformed when modeled as outcomes; raw scores were retained for descriptive analyses and correlations. Primary models adjusted for age, sex, education, and additional earthquake-related adversity, selected *a priori* as established correlates of post-disaster distress; when adversity was the predictor of interest, models adjusted for age, sex, and education. BCa bootstrap 95% confidence intervals based on 5,000 resamples were calculated for coefficients of primary interest. Model assumptions and influential observations were examined.

Because brooding and reflection were highly correlated, brooding was used as the primary rumination measure and reflection was examined in parallel analyses. Bereavement moderation of the brooding–posttraumatic stress association was tested hierarchically by adding the mean-centered brooding × bereavement interaction after covariates and main effects. Interaction coefficients were reported unstandardized. Robustness was examined using within-group standardization, exclusion of influential observations, robust bisquare M-estimation, and an alternative bereavement definition restricted to close losses. In an exploratory analysis that was not prespecified, the three common adversity types were entered as separate indicators in place of the count, and model fit was compared with a constrained equal-weight model (**Supplementary Table 2**).

The independent associations of bereavement and additional adversity with posttraumatic stress were estimated in the same model. Participants were also classified into four loss–adversity groups: neither, adversity only, bereavement only, and both. Grief-specific analyses were restricted to the 64 participants who had lost a family member or close friend. Within this subsample, hierarchical regression tested the association between meaninglessness and prolonged grief after adjustment for posttraumatic stress, depression, and other covariates. A sensitivity analysis repeated this model using a grief score excluding PG-13 items overlapping with meaning and emptiness.

No *a priori* power analysis was conducted. *Post hoc* sensitivity analyses indicated minimum detectable effects of f² = .056 for the full-sample interaction model and approximately f² = .14 for close-loss models at 80% power and α = .05. The Benjamini–Hochberg false discovery rate procedure was applied to a family of six primary analyses defined for this report.

## 3 Results

### 3.1 Sample characteristics and symptom frequency

Participants ranged in age from 18 to 56 years (M = 28.4, SD = 7.8), and 42.3% were women. Seventy-five participants (50.3%) reported an earthquake-related bereavement; 64 had lost a family member or close friend, including five who had lost a first-degree relative. HAM-D scores were ≥8 in 34.9% of the sample (95% CI [27.2, 42.6]), and 23.5% (95% CI [16.7, 30.3]) had IES-R scores ≥33. Among the 64 participants with a close loss, 10 (15.6%) met criteria for probable prolonged grief disorder (PGD); only one did so without also exceeding the threshold for either posttraumatic stress or depression.

Bereaved participants were older, reported more additional earthquake-related adversity, and had higher scores on all symptom measures than non-bereaved participants, whereas the groups did not differ in sex, education, or marital status (**Table 1**). Women had higher posttraumatic stress scores than men (IES-R M = 30.6 vs. 15.2) and remained so in adjusted models (p <.001). Within the close-loss subsample, women also had higher unadjusted grief scores (PG-13 M = 31.1 vs. 19.7), although the difference was no longer significant after adjustment for concurrent symptoms (p = .43). Internal consistency coefficients ranged from α = .82 to.97 (**Supplementary Table 1**).

**Table 1:** Sociodemographic and clinical characteristics by bereavement status.

Characteristic	Total (N = 149)	Bereaved (n = 75)	Non-bereaved (n = 74)	p
Age, M (SD)	28.4 (7.8)	30.5 (8.7)	26.2 (6.2)	<.001
Female, n (%)	63 (42.3)	34 (45.3)	29 (39.2)	.448
Education, n (%)				.763
Primary school	17 (11.4)	9 (12.0)	8 (10.8)	
High school	72 (48.3)	34 (45.3)	38 (51.4)	
University or higher	60 (40.3)	32 (42.7)	28 (37.8)	
Marital status, n (%)				.413
Never married	83 (55.7)	38 (50.7)	45 (60.8)	
Married	54 (36.2)	31 (41.3)	23 (31.1)	
Widowed/divorced	12 (8.1)	6 (8.0)	6 (8.1)	
Additional adversity count, M (SD)	0.54 (0.97)	0.93 (1.14)	0.14 (0.51)	<.001
Individual adversity types, n (%)				
Shelter problems	33 (22.1)	29 (38.7)	4 (5.4)	<.001
Difficulty obtaining food	26 (17.4)	22 (29.3)	4 (5.4)	<.001
Witnessing death	18 (12.1)	16 (21.3)	2 (2.7)	<.001
Entrapment under rubble	2 (1.3)	2 (2.7)	0 (0.0)	—
Serious injury	1 (0.7)	1 (1.3)	0 (0.0)	—
Posttraumatic stress (IES-R), M (SD)	21.7 (20.3)	29.7 (22.3)	13.6 (14.1)	<.001
Depression (HAM-D), M (SD)	6.3 (4.6)	7.1 (5.0)	5.4 (4.0)	.023
Brooding (RRS-SF), M (SD)	8.9 (3.7)	10.4 (4.3)	7.5 (2.3)	<.001
Reflection (RRS-SF), M (SD)	8.8 (3.5)	9.9 (3.8)	7.7 (2.8)	<.001

Bereaved = any earthquake-related bereavement; 64 of the 75 bereaved participants had lost a family member or close friend. Group comparisons used Welch’s t, χ², or Fisher’s exact tests and are descriptive; effect sizes are reported in **Supplementary Table 1**. The adversity count is the number of adversity types endorsed (0–5); participants could endorse more than one type, and entrapment and serious injury were too rare to test. IES-R, Impact of Event Scale–Revised; HAM-D, Hamilton Depression Rating Scale; RRS-SF, Ruminative Responses Scale–Short Form.

Additional earthquake-related adversity was relatively uncommon (M = 0.54, SD = 0.97); 106 participants (71.1%) reported none of the five adversity types. Individual frequencies ranged from 22.1% for shelter problems to 0.7% for serious injury (**Table 1**). Because entrapment under rubble and serious injury were reported by only two and one participants, respectively, a sensitivity analysis using a count restricted to the three more common adversity types is reported in Section 3.3.

### 3.2 Correlations among study variables

In the full sample, both brooding and reflection were strongly correlated with posttraumatic stress; the correlation between brooding and IES-R scores was r = .73 (**Table 2**). Within the close-loss subsample, prolonged grief was strongly associated with meaninglessness, posttraumatic stress, and brooding. Brooding and reflection were highly correlated in both the full sample (r = .85) and the close-loss subsample (r = .88) and were therefore not entered simultaneously in regression models.

**Table 2:** Correlations among study variables.

Variable	1	2	3	4	5	6
1. Age	—	.21	.15	.17	.44***	.20
2. Additional adversity count	.30***	—	.27*	.25*	.46***	.25*
3. Brooding (RRS-SF)	.25**	.44***	—	.88***	.65***	.43***
4. Reflection (RRS-SF)	.17*	.38***	.85***	—	.69***	.37**
5. Posttraumatic stress (IES-R)	.40***	.54***	.73***	.72***	—	.53***
6. Depression (HAM-D)	.21**	.30***	.46***	.36***	.46***	—
Prolonged grief (PG-13)^a^	.35**	.43***	.70***	.71***	.73***	.46***
Meaninglessness (GMRI)^a^	.32**	.32**	.53***	.50***	.73***	.50***

Values below the diagonal are from the full sample (N = 149); values above the diagonal are from the close-loss subsample (n = 64).

^a^Prolonged grief and meaninglessness were defined only in the close-loss subsample; their correlation with each other was r = .69, p <.001. PG-13, Prolonged Grief Scale; GMRI, Grief and Meaning Reconstruction Inventory.

*p <.05. **p <.01. ***p <.001.

### 3.3 Bereavement, additional adversity, and their co-occurrence

When additional earthquake-related adversity and bereavement status were entered simultaneously, both were independently associated with posttraumatic stress. The association was stronger for additional adversity (β = .38; unique ΔR² = .111) than for bereavement (β = .19; unique ΔR² = .027).

Mean symptom levels across the four loss–adversity groups are shown in **Figure 1A**. Mean IES-R scores were 12.2 (SD = 12.9; n = 68) among participants reporting neither bereavement nor additional adversity, 29.3 (SD = 18.7; n = 6) among those reporting adversity only, 16.7 (SD = 18.9; n = 38) among those reporting bereavement only, and 43.0 (SD = 17.3; n = 37) among those reporting both. Mean HAM-D scores in the same groups were 5.3, 6.8, 5.0, and 9.3, respectively.

**Figure 1:**
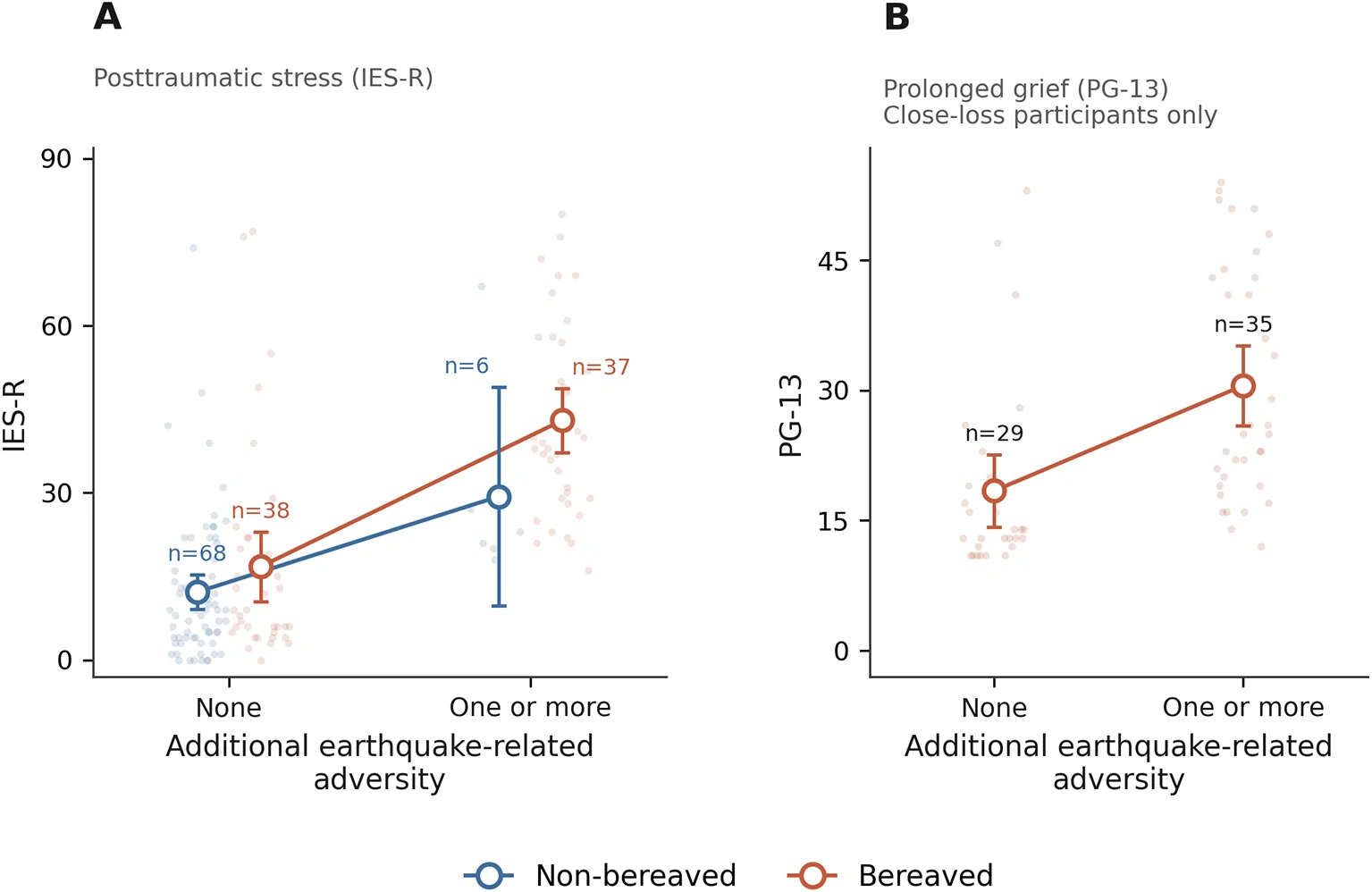
**(A, B)** Posttraumatic stress and prolonged grief by bereavement and additional earthquake-related adversity. **(A)** Shows IES-R scores among non-bereaved and bereaved participants, according to whether any additional earthquake-related adversity was reported. **(B)** Shows PG-13 scores for the same contrast among close-loss participants; grief is defined only in the presence of a loss, so a single series is shown. Faded points are individual observations; open circles are group means with 95% confidence intervals. Group sizes are annotated in the figure. The adversity-only group was small and is shown without inferential comparison. IES-R, Impact of Event Scale–Revised; PG-13, Prolonged Grief Scale.

Among bereaved participants, those who also reported additional adversity had substantially higher posttraumatic stress scores than those without additional adversity (mean difference = 26.2, 95% CI [17.9, 34.6]; Welch’s t(72.7) = 6.28, p <.001; Hedges g = 1.43, 95% CI [0.93, 1.94]). A similar pattern was observed for prolonged grief within the close-loss subsample (**Figure 1B**): participants with additional adversity had higher PG-13 scores than those without it (M = 30.5 vs. 18.4; mean difference = 12.1, 95% CI [6.0, 18.2]; t(62.0) = 3.98, p <.001; g = 0.97, 95% CI [0.45, 1.48]).

By contrast, posttraumatic stress did not differ significantly between bereaved participants without additional adversity and participants reporting neither bereavement nor adversity (M = 16.7 vs. 12.2; mean difference = 4.6, 95% CI [−2.3, 11.5]; t(56.5) = 1.32, p = .19; g = 0.30, 95% CI [−0.10, 0.69]). Because the adversity-only group was small (n = 6), no inferential pairwise comparison involving this group was performed.

Within the close-loss subsample, additional adversity remained independently associated with greater prolonged grief after adjustment for posttraumatic stress, depression, and demographic covariates (β = .24, p = .007; **Table 3**).

**Table 3:** Prediction of prolonged grief in the close-loss subsample.

Predictor	B [95% CI]	β	p
Age	0.0033 [−0.0063, 0.0129]	.06	.497
Sex (female)	0.0949 [−0.0894, 0.2793]	.09	.307
Education	−0.1892 [−0.3048, −0.0735]	−.26	.002
Additional adversity count	0.1048 [0.0301, 0.1795]	.24	.007
Posttraumatic stress (IES-R)	0.0075 [0.0013, 0.0136]	.33	.018
Depression (HAM-D)	0.0017 [−0.0163, 0.0197]	.02	.852
Meaninglessness (GMRI)	0.0239 [0.0029, 0.0448]	.25	.027

n = 64. The outcome was log-transformed PG-13. Model R² = .697, F(7, 56) = 18.37, p <.001. Hierarchical steps: demographics and additional adversity, R² = .534; adding posttraumatic stress and depression, ΔR² = .135; adding meaninglessness, ΔR² = .028 (f² = .093). BCa bootstrap 95% CIs based on 5,000 resamples were [0.0285, 0.2061] for additional adversity and [−0.0011, 0.0399] for meaninglessness. PG-13, Prolonged Grief Scale; GMRI, Grief and Meaning Reconstruction Inventory; IES-R, Impact of Event Scale–Revised; HAM-D, Hamilton Depression Rating Scale; B, unstandardized coefficient; β, standardized coefficient.

In the sensitivity analysis excluding the two rare adversity types, the three-item and five-item adversity counts were almost perfectly correlated (r = .99), and the main findings were materially unchanged: β = .37 and unique ΔR² = .110 for posttraumatic stress, g = 1.27 for the posttraumatic stress group contrast, g = 0.85 for prolonged grief, and p = .006 for adversity in the adjusted grief model. The brooding × bereavement interaction (B = −0.247) and the associations of meaninglessness (p = .035) and education (p = .002) were similarly unchanged. In an exploratory analysis, entering the three common adversity types as separate indicators gave a pattern consistent with the count for posttraumatic stress, whereas for prolonged grief the association was concentrated in witnessing death (n = 16 in the close-loss subsample); these analyses were not prespecified and are reported in **Supplementary Table 2**.

### 3.4 Repetitive negative thinking and moderation by bereavement

After adjustment for age, sex, education, and additional adversity, both brooding (β = .50) and reflection (β = .50) were strongly associated with posttraumatic stress (both p <.001). The brooding × bereavement interaction was significant (B = −0.24, SE = 0.08, p = .002, ΔR² = .025; bootstrap 95% CI [−0.43, −0.08]). Simple-slope analyses showed a stronger association between brooding and posttraumatic stress among non-bereaved participants (B = 0.47, 95% CI [0.33, 0.60]) than among bereaved participants (B = 0.22, 95% CI [0.14, 0.31]); both slopes were significant at p <.001 (**Table 4**).

**Table 4:** Prediction of posttraumatic stress: moderation of the brooding association by bereavement.

Predictor	B [95% CI]	β	p
Age	0.044 [0.014, 0.074]	.16	.004
Sex (female)	0.795 [0.310, 1.281]	.18	.001
Education	−0.016 [−0.367, 0.334]	.00	.927
Additional adversity count	0.560 [0.292, 0.828]	.25	<.001
Brooding	0.467 [0.330, 0.604]	.79	<.001
Bereaved	0.113 [−0.405, 0.632]	.03	.667
Brooding × Bereaved	−0.244 [−0.398, −0.090]	−.35	.002

N = 149. The outcome was square-root-transformed IES-R. Brooding and additional adversity were mean-centered; female = 1, bereaved = 1. Model R² = .646, F(7, 141) = 36.84, p <.001; the interaction step added ΔR² = .025 (f² = .070). Simple slopes were B = 0.467 for non-bereaved and B = 0.223 for bereaved participants, both p <.001. B, unstandardized coefficient; β, standardized coefficient; IES-R, Impact of Event Scale–Revised.

The interaction was similar in robust regression with bisquare M-estimation (B = −0.25) and remained significant after influential observations were excluded (B = −0.18, p = .023) and when bereavement was restricted to the loss of a family member or close friend (B = −0.18, p = .017), although the estimated effect was smaller in the latter two analyses. In contrast, the interaction was not retained after within-group standardization (B = −0.12, p = .61). Bereavement did not moderate the association between additional adversity and posttraumatic stress (B = −0.15, p = .67). The reflection × bereavement interaction was in the same direction but was not significant (B = −0.08, p = .30), nor was the sex × brooding interaction (p = .28). Taken together, the interaction was retained under robust estimation, after exclusion of influential observations, and when bereavement was restricted to close losses, but not after within-group standardization; it should therefore be regarded as a preliminary, sensitivity-dependent finding.

### 3.5 Prolonged grief in the close-loss subsample

Among participants with a close loss (n = 64), greater meaninglessness was associated with greater prolonged grief after adjustment for posttraumatic stress, depression, additional adversity, and demographic covariates (β = .25, ΔR² = .028, p = .027; **Table 3**). However, the BCa bootstrap 95% CI for the unstandardized coefficient included zero [−0.001, 0.040]. When PG-13 items overlapping in content with meaning and emptiness were removed from the outcome, the coefficient was smaller and not statistically significant (β = .20, p = .12). The incremental effect observed in the primary model (f² = .093) was smaller than the minimum effect detectable with 80% power in this subsample (approximately f² = .140). The non-significant result of this sensitivity analysis should therefore not be interpreted as evidence of no association, but rather as an imprecise estimate in a sample with limited power to detect effects of this magnitude.

In the same model, higher educational attainment was independently associated with lower prolonged grief severity (β = −.26, p = .002), whereas depression was not (p = .85). Brooding was associated with prolonged grief after adjustment for depression (β = .41, p <.001); however, the difference between the brooding–grief and brooding–depression coefficients was not significant (Δβ = .19, 95% CI [−0.12, 0.53]).

All effects that were significant in the six primary analyses defined for this report remained significant after Benjamini–Hochberg false discovery rate correction. The adjusted p value was.027 for the incremental association of meaninglessness and.021 for the association between posttraumatic stress and prolonged grief. Neither met the more conservative Bonferroni threshold of α = .0083.

## 4 Discussion

Approximately three years after the 2023 Kahramanmaraş earthquakes, symptom burden was substantial in this clinical sample. About one third of participants had elevated clinician-rated depressive symptoms, 23.5% met the screening threshold for posttraumatic stress, and 15.6% of those with a close loss met criteria for probable PGD. The clearest pattern was observed among participants who experienced both bereavement and additional earthquake-related adversity, in whom both posttraumatic stress and grief severity were markedly elevated. By contrast, the difference in posttraumatic stress between participants with bereavement alone and those with neither bereavement nor additional adversity was modest and not statistically significant. Two further findings were sensitive to analytic choices and should be regarded as preliminary. The association between repetitive negative thinking and posttraumatic stress was weaker among bereaved participants, but this difference was not retained after within-group standardization. Meaninglessness showed an incremental association with grief beyond posttraumatic stress and depression, but the estimate was imprecise and attenuated when overlapping grief items were removed.

### 4.1 Co-occurrence of bereavement and additional adversity

Among bereaved participants, those who had also experienced adversities such as witnessing death, food deprivation, or loss of shelter had posttraumatic stress levels more than 2.5 times as high as those who had not experienced these additional adversities; the difference in grief severity approached one standard deviation. Both findings were retained when the alternative definition of the adversity count was used. A similar pattern was reported after the 2004 tsunami: bereaved individuals who had been present in the disaster area showed higher levels of prolonged grief and posttraumatic stress than bereaved relatives who had not been directly exposed (14), and the combination of trauma and loss was associated with higher rates of comorbidity (13). Findings across studies, however, have not been entirely consistent. In another tsunami cohort, direct exposure was not associated with complicated grief (15). One possible explanation is variation in how exposure is defined; simply being present in the disaster area is a broader measure than the concrete adversity indicators used in the present study. The relevance of these concrete adversities is also reflected in qualitative data from the same earthquakes: in an analysis of social media posts from the weeks after the disaster, calls for basic needs, particularly shelter and food, formed one of the main themes (39). Meta-analytic findings are also consistent with the possibility that the nature of the loss and the surrounding traumatic context matter: unnatural deaths have been associated with a greater burden of prolonged grief (40), and traumatic exposure has been linked to more severe grief among people bereaved by violent loss (41).

Several explanations have been proposed for the association between additional adversity and more severe grief. Network studies have suggested that grief symptoms after earthquake-related loss may be more strongly interconnected than grief following illness-related death, with difficulties in interpersonal trust and yearning becoming more central (42). Another possibility is that the usual processes of mourning are disrupted by disaster conditions. In a qualitative study of people bereaved in the same earthquakes, inability to reach or see the deceased and difficulties with burial were associated with more severe grief and greater meaninglessness (43). These processes were not measured directly in the present study, however, and the composite count does not by itself indicate which specific adversity contributed most to the observed association.

Three limitations are important when interpreting this finding. First, because grief cannot be assessed in the absence of bereavement, the design does not allow the associations of bereavement and additional adversity to be fully separated across both outcomes. Second, the lack of a statistically significant difference in posttraumatic stress between participants with bereavement alone and those with neither bereavement nor additional adversity should not be taken to mean that bereavement was unimportant; the confidence interval remained compatible with an effect of moderate magnitude. Third, the adversity count represents the number of reported adversity types and does not weight them by severity, so that experiences of potentially different clinical significance, such as witnessing death and shelter problems, contribute equally to the primary analyses. The measure should therefore be understood as a simple count of reported additional adversities rather than as an index of trauma severity. An exploratory analysis by adversity type suggested that, for grief, the association was concentrated in witnessing death, whereas for posttraumatic stress the count and the separate indicators performed similarly (**Supplementary Table 2**). Given the small number of participants who witnessed death and the *post hoc* nature of this analysis, we retained the prespecified count as the primary measure and regard the type-specific pattern as a question for future work. The conclusion should therefore remain narrow: posttraumatic stress was more strongly associated with additional adversity than with bereavement, and both posttraumatic stress and grief severity were highest when bereavement and additional adversity co-occurred. This pattern is consistent with the view that posttraumatic stress and prolonged grief are related but distinct constructs (11).

### 4.2 Repetitive negative thinking across bereavement status

Brooding and reflection were both strongly associated with posttraumatic stress, consistent with previous meta-analytic findings (19, 20). More notably, this association was substantially weaker among participants who reported bereavement, although this difference was not retained across all sensitivity analyses. Meta-analytic work has suggested that the association between rumination and posttraumatic stress is relatively stable across a range of moderators (20). In the present study, however, the brooding–posttraumatic stress association was reduced by approximately half among bereaved participants, whereas bereavement did not similarly modify the association between additional adversity and posttraumatic stress. This pattern suggests that bereavement may alter the strength of the association between repetitive negative thinking and posttraumatic stress. Prospective findings showing that this association can vary across trauma types are also consistent with this possibility, although that study measured trauma-specific rumination and observed moderation only for numbing symptoms (21).

This finding should nevertheless be interpreted cautiously. After within-group standardization, conducted to account for the narrower distribution of brooding scores in the non-bereaved group, the interaction was no longer significant (B = −0.12, p = .61). It was retained under robust estimation, after exclusion of influential observations, and under an alternative bereavement definition restricted to close losses, although the coefficient was smaller in the latter two analyses. The moderation should therefore be regarded as sensitive to how brooding is scaled. In addition, brooding and reflection were highly correlated, so the observed moderation cannot be considered specific to brooding alone; it is more appropriate to interpret the finding in terms of repetitive negative thinking more broadly. Other explanations are also possible. Among bereaved participants, more of the variation in posttraumatic stress may be tied to additional earthquake-related adversity itself, leaving less room for a general cognitive style to show an association, and repetitive thoughts after loss may focus on grief-specific content that is not fully captured by general brooding items (44–46). Rumination during bereavement has also been proposed to serve an avoidant function (22, 23). We therefore regard the moderation as a preliminary observation rather than an established effect. Replication using grief-specific measures of rumination, ideally with repeated assessments that can establish temporal order, is needed.

### 4.3 Meaninglessness, brooding, and education

Meaninglessness remained associated with prolonged grief after adjustment for posttraumatic stress and clinician-rated depression. This finding is consistent with meaning reconstruction models (24) and with prospective studies, using the same inventory, that link difficulty making sense of a loss to subsequent prolonged grief symptoms (47, 48). Three observations temper this finding: the bootstrap confidence interval included zero, the coefficient was smaller and non-significant after overlapping PG-13 items were removed, and the observed effect was below the minimum detectable effect in this subsample. The result may partly reflect shared item content, but a small independent association also cannot be excluded. We therefore regard this finding as preliminary. At the same time, meaninglessness has been identified as a central feature in symptom networks of prolonged grief (26), so removing meaning-related grief items may also exclude a clinically important part of the construct itself. Park’s distinction between meaning-making efforts and meaning made may provide a useful framework for future work; the GMRI subscale used here primarily captures the latter (49).

Brooding was also associated with prolonged grief after adjustment for depression. However, the brooding–grief association was not significantly stronger than the brooding–depression association. Brooding is therefore better understood here as a correlate shared across grief and depression rather than as a grief-specific feature, consistent with the transdiagnostic nature of repetitive negative thinking (50).

Higher educational attainment was independently associated with lower grief severity, and this association remained after adjustment for additional adversity, posttraumatic stress, and depression. A similar association was reported after the 2004 tsunami, where lower education predicted complicated grief among bereaved survivors (13). Education may reflect broader differences in material and informational resources, access to health care, or pre-earthquake socioeconomic conditions. Differences in response style may also have contributed. The finding is consistent with studies highlighting the association of socioeconomic factors with mental health outcomes after the same disaster (2), but the present dataset did not include sufficiently detailed socioeconomic measures to clarify the underlying mechanism.

### 4.4 Clinical implications

The most important clinical implication concerns long-term assessment. Approximately three years after the earthquakes, the highest symptom burden was observed among participants who had experienced both bereavement and additional earthquake-related adversity. In long-term psychosocial support programs, this pattern supports a two-part initial inquiry: whether the person lost someone in the disaster, and which concrete adversities they experienced, including witnessing death, loss of shelter, and difficulty meeting basic needs. Survivors reporting both could then be prioritized for fuller assessment. Because prolonged grief was not fully accounted for by posttraumatic stress or depression, assessment should also include a grief-specific measure rather than relying on trauma or depression screening alone. These suggestions are based on the pattern observed in this sample; the study did not evaluate the sensitivity, specificity, or predictive performance of any screening rule, and they should therefore be tested prospectively before being adopted as protocol.

Probable PGD in this sample usually co-occurred with elevated posttraumatic stress or depression, yet grief severity showed associations that were not fully accounted for by these symptoms. Similar overlap has been reported in trauma-exposed treatment-seeking samples, in which high levels of prolonged grief symptoms co-occurred with posttraumatic stress and depression but were not reducible to either (51).

A second implication concerns the persistence of symptoms. The proportion of participants scoring above the probable-PTSD threshold was similar to rates reported during earlier phases after the same disaster (52, 53). A recent study of 479 teachers working in Kahramanmaraş, surveyed during the 2024–2025 academic year, found that 34.4% were classified as having high post-earthquake trauma levels on the Post-Earthquake Trauma Level Scale, with higher trauma scores moderately associated with lower psychological resilience (54). The proportion meeting probable PGD criteria was also comparable to rates reported years after other earthquakes (55, 56), somewhat higher than the 10–11% found in Turkish community samples of bereaved adults (28), and close to the approximately 13% average reported across bereaved samples from 16 countries (6). Direct comparison is limited by differences in samples and measurement methods, but these findings suggest that clinically relevant symptoms may persist for years. After the 2004 tsunami, psychiatric morbidity likewise showed little decline between two and six years after bereavement (3).

Meta-analytic evidence indicates that grief-focused cognitive behavioral therapy can reduce prolonged grief symptoms (57), although in a recent randomized trial its advantage over an active comparator was limited to comorbid symptoms (58). By contrast, a meta-analysis of interventions delivered after the 2023 Türkiye earthquakes found that studies focused on trauma, depression, or anxiety, with grief not directly targeted (8, 59). Grief-specific interventions should therefore be considered within longer-term disaster mental health services. Cultural context is particularly relevant. In Türkiye, Islamic mourning rituals may structure the early grieving process and mobilize social support (60), while disruption of these rituals after mass-casualty earthquakes has been associated with more difficult grieving experiences (43).

### 4.5 Strengths and limitations

Strengths of the study include the use of clinician-rated depression, restriction of grief-specific analyses to participants with a close loss, and evaluation of the main findings across several alternative models and sensitivity analyses.

Several limitations should be considered. First, the cross-sectional design precludes temporal or causal inference. A single assessment three years after the earthquakes cannot distinguish symptoms that have persisted since the disaster from those that emerged or worsened later, nor can it identify resilient, recovering, or chronic trajectories (61). The direction of the association between repetitive negative thinking and posttraumatic stress is likewise uncertain; cognitive processes measured at this point may themselves have been shaped by concurrent symptoms. Furthermore, additional adversity and characteristics of the loss were reported retrospectively, approximately three years after the events. Participants with greater current symptom severity may recall or report adverse experiences more readily, which could inflate associations between reported adversity and posttraumatic stress or grief. This concern applies most directly to the higher symptom levels observed among bereaved participants who also reported additional adversity, but less to bereavement status itself, which is less susceptible to recall bias. Prospective assessment of disaster exposure would be needed to address this limitation.

Second, the sample was drawn from a single clinical center and was heterogeneous with respect to reason for presentation. Based on retrospective estimates by the assessing psychiatrists, approximately one third of participants attended for a medical-board assessment; however, reason for presentation was not recorded at the individual level and could therefore not be examined or adjusted for. Other participants presented for an initial psychiatric evaluation or ongoing outpatient treatment. Psychiatric diagnoses not covered by the exclusion criteria, treatment status, and psychotropic medication use were also not systematically recorded. These features limit interpretation in two ways. First, the frequencies of elevated depressive, posttraumatic stress, and grief symptoms describe this clinical sample and should not be interpreted as population prevalence estimates. The direction of any resulting bias is uncertain: the context of a medical-board assessment may encourage over-reporting of symptoms, whereas ongoing treatment may reduce symptom severity. Second, if reasons for presentation were unevenly distributed across the bereavement and adversity groups, some of the observed group differences could reflect clinical context rather than the stressors themselves; adjustment for demographic variables and additional adversity cannot account for this possibility. The number of eligible individuals who declined participation further raises the possibility of selection bias.

Third, bereaved and non-bereaved participants differed in age, additional adversity, and overall symptom burden. Statistical adjustment reduces but cannot eliminate confounding by unmeasured factors.

Fourth, additional adversity was measured with a single, unvalidated multiple-response item, and the resulting count captures the number of adversity types rather than their severity. Serious injury and entrapment under rubble were rare, so the measure primarily reflected witnessing death, difficulty obtaining food, and shelter problems; the findings should therefore not be generalized to severe physical trauma. Although analyses using a three-item count that excluded the two rare physical exposures produced materially similar results, this does not substitute for a validated measure of disaster exposure or trauma severity. Because adversities were reported by marking options on a single item, some experiences may also have gone unreported. The retrospective nature of these reports is discussed above.

Fifth, several potentially relevant factors were not assessed, including pre-loss symptoms, anxious attachment (62), social support (63), grief-specific rumination, religious coping, disruption of mourning rituals, and socioeconomic conditions. Sixth, grief-specific analyses were limited to 64 participants, only five of whom had lost a first-degree relative; this limits both generalizability to those bereaved of a first-degree relative and power to detect small effects.

Finally, probable PTSD and PGD were identified using screening thresholds and an algorithm rather than structured diagnostic interviews. The strong correlation between brooding and posttraumatic stress also leaves open some contribution of shared method variance across self-report measures, although depression was clinician-rated and not subject to the same method.

## 5 Conclusions

Three years after the 2023 Türkiye earthquakes, symptom burden was substantial in this clinical sample and was highest among participants who had experienced both bereavement and additional earthquake-related adversity. Among bereaved participants, additional adversity was associated with higher posttraumatic stress and more severe grief, and its association with grief remained after adjustment for posttraumatic stress and depression. Bereavement without additional adversity was associated with only a modest, statistically non-significant difference in posttraumatic stress, although a moderate effect could not be excluded. Higher educational attainment was associated with lower grief severity. Two further findings, the weaker association between repetitive negative thinking and posttraumatic stress among bereaved participants and the association between meaninglessness and grief, were sensitive to analytic choices and should be regarded as preliminary. For long-term post-disaster assessment, these findings support asking about both bereavement and concrete adversities and assessing grief separately from posttraumatic stress and depression. Longitudinal studies using diagnostic interviews and validated exposure measures are needed.

## Data Availability

The datasets presented in this article are not readily available because they contain information that could compromise the privacy of participants assessed in a clinical setting. Deidentified data may be made available for legitimate research purposes upon reasonable request and subject to approval by the local ethics committee. Requests to access the datasets should be directed to the corresponding author.
