# Supplementary material for: Loss plus adversity: posttraumatic stress and prolonged grief three years after the 2023 Türkiye earthquakes
Source: Front Psychiatry. 2026 Sep 28;17:1983790. doi: 10.3389/fpsyt.2026.1983790 (PMC13655132; doi:10.3389/fpsyt.2026.1983790)
Supplement: Supplementary file 1 [file Table1.docx]

**Supplemental Materials**

**Supplemental Table 1. Descriptive Statistics, Internal Consistency, and Effect Sizes for Group Comparisons**

| **Measure** | **k** | **M (SD)** | **Observed range** | **α** | **Bereaved vs. non-bereaved, g [95% CI]** |
| --- | --- | --- | --- | --- | --- |
| Age (years) | — | 28.4 (7.8) | 18–56 | — | 0.58 [0.25, 0.90] |
| Additional adversity count | 5 | 0.54 (0.97) | 0–4 | — | 0.90 [0.56, 1.23] |
| Posttraumatic stress (IES-R) | 22 | 21.7 (20.3) | 0–80 | .97 | 0.86 [0.52, 1.19] |
| Depression (HAM-D) | 17 | 6.3 (4.6) | 0–18 | —ᵇ | 0.38 [0.05, 0.70] |
| Brooding (RRS-SF) | 5 | 8.9 (3.7) | 5–20 | .90 | 0.82 [0.49, 1.15] |
| Reflection (RRS-SF) | 5 | 8.8 (3.5) | 5–20 | .86 | 0.66 [0.33, 0.99] |
| Prolonged grief (PG-13)ᵃ | 11 | 25.0 (13.7) | 11–54 | .96 | — |
| Meaninglessness (GMRI)ᵃ | 6 | 14.6 (5.5) | 6–28 | .82 | — |

*Note.* N = 149 unless otherwise indicated. k = number of items. Effect sizes are bias-corrected Hedges g with 95% confidence intervals for the group comparisons reported in Table 1. For categorical variables, Cramér's V was .06 for sex, .06 for education, and .11 for marital status.

ᵃ Computed in the close-loss subsample (n = 64); no group comparison is defined because these measures presuppose a loss. ᵇ The HAM-D was clinician-rated and only the total score was retained, precluding estimation of internal consistency. IES-R = Impact of Event Scale–Revised; HAM-D = Hamilton Depression Rating Scale; RRS-SF = Ruminative Responses Scale–Short Form; PG-13 = Prolonged Grief Scale; GMRI = Grief and Meaning Reconstruction
