# Supplementary material for: Loss plus adversity: posttraumatic stress and prolonged grief three years after the 2023 Türkiye earthquakes
Source: Front Psychiatry. 2026 Sep 28;17:1983790. doi: 10.3389/fpsyt.2026.1983790 (PMC13655132; doi:10.3389/fpsyt.2026.1983790)
Supplement: Supplementary file 2 [file Table2.docx]

**Supplemental Table 2. Exploratory Associations of Individual Adversity Types With Posttraumatic Stress and Prolonged Grief**

| **Adversity type** | **B (SE)** | **β** | **p** | **BCa 95% CI for B** |
| --- | --- | --- | --- | --- |
| **Panel A. Posttraumatic stress (√IES-R), full sample (N = 149)** |  |  |  |  |
| Witnessing death | 1.30 (0.51) | .19 | .012 | [−0.03, 2.61] |
| Difficulty obtaining food | 1.04 (0.49) | .18 | .034 | [0.20, 1.79] |
| Shelter problems | 0.40 (0.47) | .08 | .391 | [−0.42, 1.02] |
| Serious injury and/or entrapment under rubble | 0.48 (1.00) | .03 | .629 | — |
| Model R² | .478 |  |  |  |
| Constrained count model R² | .473 |  |  |  |
| Difference in fit | F(2, 140) = 0.75 |  | .474 |  |
| **Panel B. Prolonged grief (log PG-13), close-loss subsample (n = 64)** |  |  |  |  |
| Witnessing death | 0.38 (0.11) | .32 | .001 | [0.16, 0.63] |
| Difficulty obtaining food | 0.07 (0.10) | .06 | .498 | [−0.18, 0.27] |
| Shelter problems | −0.04 (0.10) | −.04 | .701 | [−0.24, 0.15] |
| Serious injury and/or entrapment under rubble | −0.04 (0.18) | −.02 | .826 | — |
| Model R² | .734 |  |  |  |
| Constrained count model R² | .697 |  |  |  |
| Difference in fit | F(2, 53) = 3.57 |  | .035 |  |

Note. In each panel, the three common adversity types were entered as separate binary indicators, while serious injury and entrapment under rubble were combined into a single rare-adversity indicator because of their low frequency. Panel A was adjusted for age, sex, education, and bereavement status; Panel B was adjusted for age, sex, education, posttraumatic stress, depression, and meaninglessness, consistent with Table 4. Model fit was compared with a constrained model in which the three common adversity types were represented by an equal-weight count while the rare-adversity indicator was retained separately (2 df). BCa confidence intervals were based on 5,000 resamples. For witnessing death in Panel A, the conventional p value was significant, whereas the BCa 95% CI included zero; this estimate should therefore be interpreted cautiously. BCa intervals were not reported for the rare-adversity indicator because only three participants endorsed this category. Frequencies are reported as n in the full sample (n in the close-loss subsample): witnessing death, 18 (16); difficulty obtaining food, 26 (21); shelter problems, 33 (27); serious injury or entrapment, 3 (3). The three common adversity types were moderately intercorrelated (φ = .45–.65). B = unstandardized coefficient; β = standardized coefficient; IES-R = Impact of Event Scale–Revised; PG-13 = Prolonged Grief Scale.
